# Peak left atrial longitudinal strain is associated with all-cause mortality in patients with ventricular functional mitral regurgitation

**DOI:** 10.1186/s12947-023-00307-7

**Published:** 2023-05-06

**Authors:** Daniel A. Gomes, Pedro M. Lopes, Pedro Freitas, Francisco Albuquerque, Carla Reis, Sara Guerreiro, João Abecasis, Marisa Trabulo, António M. Ferreira, Jorge Ferreira, Regina Ribeiras, Miguel Mendes, Maria J. Andrade

**Affiliations:** Department of Cardiology, Hospital de Santa Cruz, Centro Hospitalar de Lisboa Ocidental, Av. Prof. Dr. Reinaldo Dos Santos, 2790-134 Carnaxide, Lisbon, Portugal

**Keywords:** Functional mitral regurgitation, Peak atrial longitudinal strain, Echocardiography, Heart failure, Prognosis

## Abstract

**Purpose:**

Chronic mitral regurgitation promotes left atrial (LA) remodeling. However, the significance of LA dysfunction in the setting of ventricular functional mitral regurgitation (FMR) has not been fully investigated. Our aim was to assess the prognostic impact of peak atrial longitudinal strain (PALS), a surrogate of LA function, in patients with FMR and reduced left ventricular ejection fraction (LVEF).

**Methods:**

Patients with at least mild ventricular FMR and LVEF < 50% under optimized medical therapy who underwent transthoracic echocardiography at a single center were retrospectively identified in the laboratory database. PALS was assessed by 2D speckle tracking in the apical 4-chamber view and the study population was divided in two groups according to the best cut-off value of PALS, using receiver operating characteristics (ROC) curve analysis. The primary endpoint-point was all-cause mortality.

**Results:**

A total of 307 patients (median age 70 years, 77% male) were included. Median LVEF was 35% (IQR: 27 – 40%) and median effective regurgitant orifice area (EROA) was 15mm^2^ (IQR: 9 – 22mm^2^). According to current European guidelines, 32 patients had severe FMR (10%). During a median follow-up of 3.5 years (IQR 1.4 – 6.6), 148 patients died. The unadjusted mortality incidence per 100 persons-years increased with progressively lower values of PALS. On multivariable analysis, PALS remained independently associated with all-cause mortality (adjusted hazard ratio 1.052 per % decrease; 95% CI: 1.010 – 1.095; *P* = 0.016), even after adjustment for several (*n* = 14) clinical and echocardiographic confounders.

**Conclusion:**

PALS is independently associated with all-cause mortality in patients with reduced LVEF and ventricular FMR.

**Graphical Abstract:**

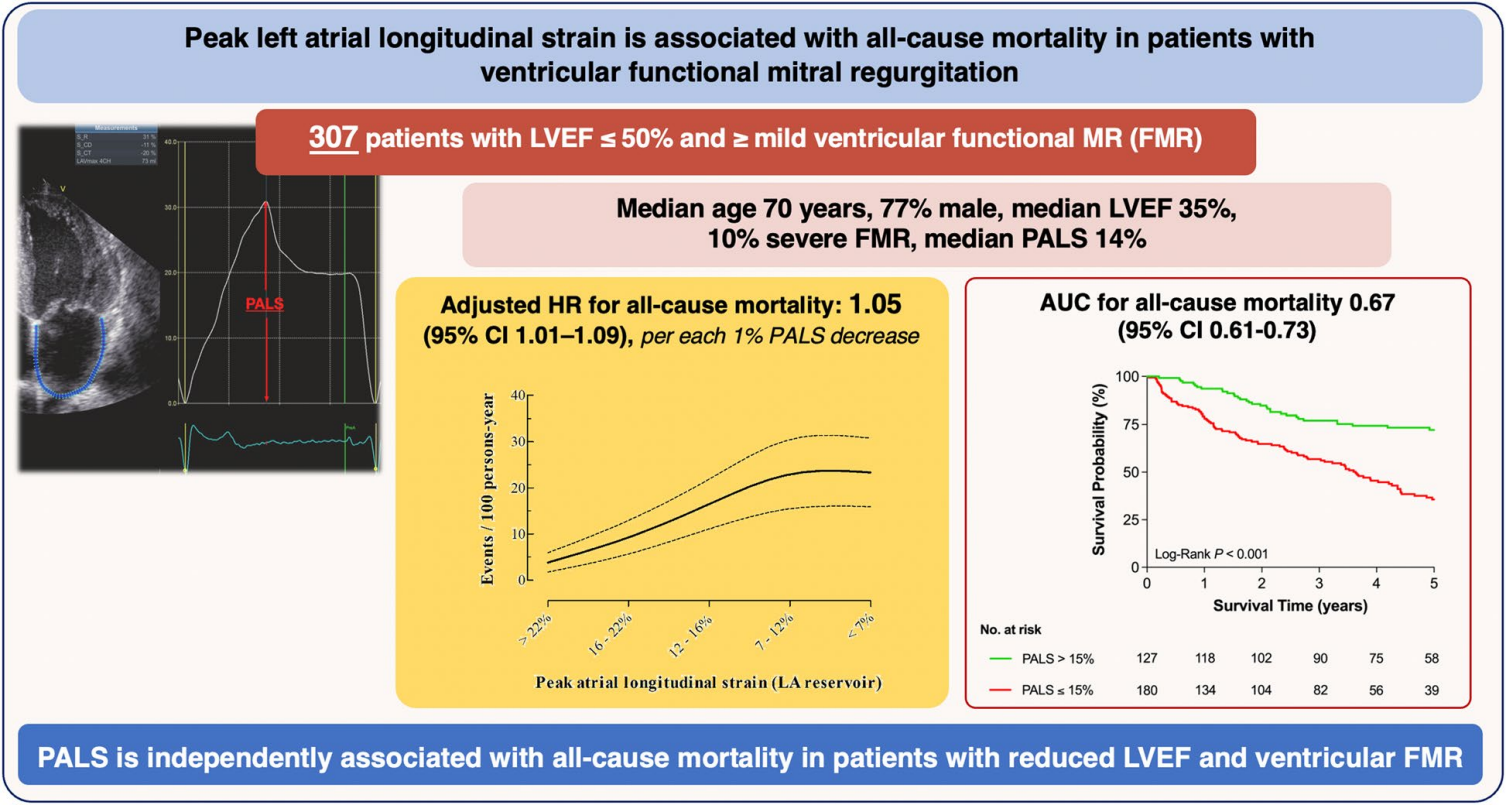

**Supplementary Information:**

The online version contains supplementary material available at 10.1186/s12947-023-00307-7.

## Introduction

Functional mitral regurgitation (FMR) is a complex and heterogeneous condition, most frequently secondary to left ventricular (LV) remodeling and dysfunction [1, 2]. It is a common finding in patients with chronic heart failure (HF) and has a strong negative impact on clinical outcomes [3, 4].

While many studies focused primarily on LV function and geometry, the interest in the left atrium (LA) has been resurging over the recent years [5]. Both pressure and volume overload associated with LV dysfunction and mitral regurgitation can produce significant structural changes, resulting in progressive dilatation [6]. In fact, growing evidence on HF suggests that LA size and function associates closely with disease progression [7, 8]. More recently, severe mitral regurgitation has been shown to promote remarkable LA ultrastructural changes and remodeling, even before dilatation occurs [7, 9–11]. Notwithstanding, the significance of LA dysfunction in the setting of LV dysfunction and ventricular FMR has not been fully investigated.

**Fig. 1:**
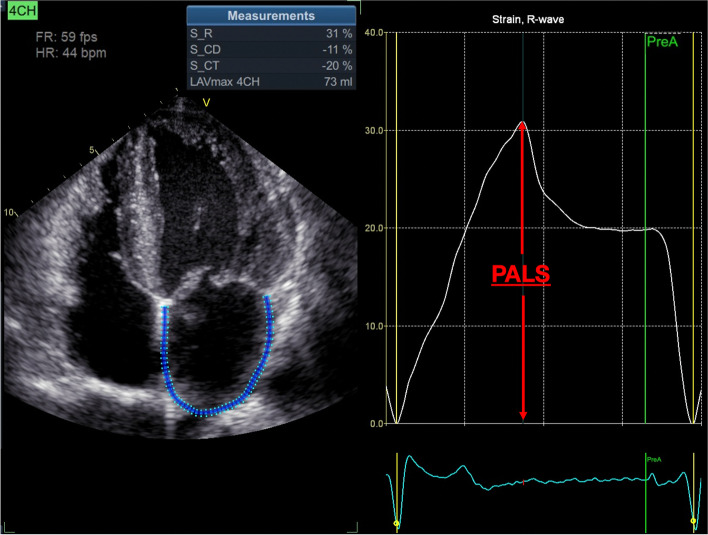
Peak atrial longitudinal strain (PALS). Legend – Measurement of peak atrial longitudinal strain (PALS) using the speckle tracking echocardiography from an apical 4-chamber view in a representative subject

2-D speckle tracking echocardiography (2D STE) is an angle-independent method for the quantification of myocardial deformation, and its feasibility for the study of LA function has been validated in previous studies [10–13]. It has showed to accurately characterize LA mechanics throughout the cardiac cycle (Fig. 1): (i) reservoir phase (atrial relaxation during ventricular systole); (ii) conduit phase (passive emptying during early diastole); and (iii) booster pump phase (atrial contraction) [12–15].

Albeit standardized reference values are still lacking, peak atrial longitudinal strain (PALS), or LA longitudinal strain during the reservoir phase, has been consistently reported as a reproducible measurement and with encouraging results [12]. It provides additive diagnostic and prognostic value to LA indexed volume in LV diastolic dysfunction and atrial fibrillation [16–18], and correlates closely with the degree of atrial fibrosis and stiffness [19].

The aim of this study was to assess the prognostic significance LA function, as assessed by PALS, in patients with LV systolic dysfunction and ventricular functional mitral regurgitation.

## Materials and methods

### Study population and clinical data

Patients with at least mild ventricular FMR and reduced left ventricle ejection fraction (LVEF), defined as LVEF < 50%, who underwent transthoracic echocardiography between 2010 and 2018 were retrospectively identified at a single center laboratory database. Optimal guideline directed medical therapy (GDMT) for at least 3 months (including implantable cardioverter defibrillator, cardiac resynchronization therapy and coronary revascularization when indicated) was a prerequisite for inclusion [20]. To isolate the effect of FMR certain exclusion criteria were applied: 1) age < 18 years; 2) at least moderate aortic stenosis or regurgitation; 3) previous valvular intervention; 4) hypertrophic cardiomyopathy; 5) arrhythmogenic right ventricular cardiomyopathy; 6) left ventricular non-compaction cardiomyopathy; 7) renal transplant patients. Patients in whom echocardiographic data was not analyzable using 2D STE were also excluded (Supplementary Fig. 1).

The first available echocardiographic study after optimal GDMT with the patient in a hemodynamically stable state served as the index time point for time-to-event analyses. Medical history and current medication were collected at the time of the index echocardiographic exam. The study protocol was reviewed and approved by the local ethics' committee, which waived the need for informed consent.

### Echocardiographic evaluation

Transthoracic echocardiography (TTE) was performed with commercially available equipment (Vivid 7 and Vivid E95, GE-Healthcare®, Chicago, Illinois). Two-dimensional (2D), M-mode and Doppler data were retrospectively retrieved from the echocardiographic database for offline analysis (EchoPAC v204, GE-Healthcare®). LV volumes (end-systolic and end-diastolic) were measured in the apical 2- and 4-chamber views and LVEF was calculated according to Simpson’s biplane method and indexed for body surface area [21]. Lateral peak early diastolic mitral annular velocities were measured with tissue Doppler imaging. As a surrogate of LV filling pressures, the ratio between peak early diastolic transmitral flow velocity and peak early diastolic mitral annular tissue velocity was calculated. LA volumes were calculated by the biplane method of disks and indexed to body surface area [21]. Right ventricular (RV) function was assessed by the tricuspid annular plane systolic excursion (TAPSE) in the apical 4-chamber view [21]. Tricuspid regurgitation was evaluated with continuous wave Doppler and systolic pulmonary artery pressures (SPAP) were estimated when possible.

Mitral regurgitation (MR) quantification was performed with the proximal isovelocity surface area (PISA) method including the effective regurgitant orifice area (EROA) and the regurgitant volume (RegVol) [22]. A 4-chamber zoomed view of the MR jet with a baseline downward shift was used to assess the hemispheric shell of the proximal flow convergence. Measurement of proximal flow convergence radius was performed at the same time point as the peak velocity during the regurgitant phase. MR severity was graded according to current European Society of Cardiology (ESC) guidelines [23].

LA speckle-tracking strain was measured using a dedicated LA-tracking software (EchoPAC v204, GE-Healthcare®) on the apical four-chamber view at a frame rate > 50fps (mean 55fps), according to current European Association of Cardiovascular Imaging (EACVI) recommendations [24]. The onset of the QRS-complex was used as the zero-reference point. The LA endocardial border was traced manually and corrected by adjusting the region of interest or the width of the contour, excluding the pulmonary vein ostia and LA appendage. The PALS was defined as the peak LA strain during the cardiac cycle. PALS was chosen over LA conduit strain and LA contractile strain because it is the LA strain parameter which has the largest body of evidence supporting its prognostic utility and can be measured in patients with atrial fibrillation [25].

### Clinical follow-up and endpoint definition

Patients were assessed at least once a year and vital status was documented at the institutional electronic medical records, which is updated using the Portuguese National Patient Registry. During follow-up, titration of medical therapy, indications for cardiac devices and any kind of mitral intervention or heart transplant/left ventricular assist device were left at the discretion of the Heart Failure Team. The primary endpoint was all-cause mortality. Vital status was available for all patients.

### Statistical analysis

Categorical values are presented as absolute numbers (and percentage) and continuous variables as mean ± standard deviation (normal distribution) or as median and interquartile range (IQR; nonparametric). Pearson’s Chi-squared (χ^2^) test, Mann–Whitney U and independent samples t-test were applied for comparison where appropriate.

The inter-observer variability of PALS measurement was assessed by calculating the intraclass correlation coefficient analysis on a random sample of 30 echocardiograms, by comparing a repeated analysis performed one week later by a second blinded independent operator. It showed very good reproducibility inter-observer variability with an intraclass correlation coefficient of 0.98 (95% CI, 0.96 – 0.99; *P* < 0.001).

The receiver operating characteristic (ROC) curve was used to determine the discriminative ability of PALS for all-cause mortality and the maximum Youden’s index was employed to identify the best cut-off of PALS. In a subsequent analysis, the population was divided into four groups according to the determined PALS cut-off and the presence of severe LA dilatation, based on current recommendations [21]. Additionally, patients were also divided into four groups according to the determined cut-off of PALS and a history of atrial fibrillation (AF).

Kaplan–Meier survival curves were plotted to compare groups, and the log-rank test was used to assess for significant differences in time to endpoint between groups. Patients were right-censured if mitral intervention (transcatheter edge-to-edger repair or surgical repair or replacement) or heart transplant/left ventricular assist device (LVAD) were performed. Cox proportional hazards regression was applied for univariable and multivariable analysis to investigate the association between clinical and echocardiographic parameters with all-cause mortality. Variables with a *P-*value < 0.05 were included in the multivariable model. Multicollinearity was excluded using variance inflation factor. The proportional hazards assumption using Schoenfeld residuals was tested and satisfied.

Statistical significance was set at *P-*value < 0.05 (two-sided). All analyses were performed using SPSS v26.0 (IBM Corporation, Armonk, New York) and STATA v13.0 (StataCorp, College Station, Texas).

## Results

### Patient population

A total of 307 patients (median age 70 years [IQR 61–77]; 77% male) were included. Baseline clinical and echocardiographic characteristics are shown in Tables 1 and 2. Most patients were in New York Heart Association (NYHA) functional classes II and III [177 (57.7%) and 105 (34.2%), respectively], and 186 patients (60.6%) had an ischemic etiology. Regarding optimal GDMT, 284 (92.5%) received beta-blockers, 284 (92.5%) angiotensin converting-enzyme inhibitors or angiotensin receptor blockers, 146 (47.8%) mineralocorticoid receptor antagonists, 87 (28.3%) had an implantable cardioverter defibrillator and 83 (27.0%) were under cardiac resynchronization therapies. Median LVEF was 35% (IQR 27–40%) and median effective regurgitant orifice area (EROA) was 15mm^2^ (9-22mm^2^). According to the new ESC 2021 guidelines for the management of valvular heart disease, 32 patients (10.4%) had severe FMR.

**Table 1:** Clinical characteristics at baseline

	**Total population (*n* = 307)**	**PALS > 15% (*n* = 127)**	**PALS ≤ 15% (*n* = 180)**	***P*-value**
**Clinical characteristics**				
Age, years	70 (62–77)	67 (57–75)	73 (65–78)	**0.001**
Male	236 (76.9)	90 (70.9)	146 (81.1)	**0.036**
BSA, m^2^	1.81 (1.71–1.94)	1.84 (1.71–1.97)	1.81 (1.70–1.92)	0.174
Atrial fibrillation	133 (43.3)	34 (26.8)	99 (55.0)	** < 0.001**
Hypertension	225 (73.3)	92 (72.4)	133 (73.9)	0.778
Diabetes mellitus	93 (30.3)	29 (22.8)	64 (35.6)	**0.017**
Creatinine, mg/dL	1.15 (0.91–1.78)	0.99 (0.84–1.40)	1.29 (1.02–1.93)	** < 0.001**
EuroSCORE II, %	4.21 (2.35–78)	2.66 (1.70–4.98)	5.39 (3.38–9.20)	** < 0.001**
NYHA functional class				** < 0.001**
I	22 (7.2)	16 (12.6)	6 (3.3)	
II	177 (57.7)	89 (70.1)	88 (48.9)	
III	105 (34.2)	22 (17.3)	83 (46.1)	
IV	3 (1.0)	0 (0.0)	3 (1.7)	
Heart failure etiology				0.823
Ischemic	186 (60.6)	76 (59.8)	110 (61.1)	
Nonischemic	121 (39.4)	51 (40.2)	70 (38.9)	
**Medication and devices**				
ACEi/ARB	284 (92.5)	120 (94.5)	164 (91.1)	0.269
percent of maximal dose, %	75 (25–100)	100 (50–100)	50 (25–100)	** < 0.001**
Beta-blockers	284 (92.5)	118 (92.9)	166 (92.2)	0.821
percent of maximal dose, %	50 (25–100)	50 (25–100)	50 (25–75)	0.378
MRA	146 (47.8)	50 (39.4)	96 (53.3)	**0.016**
percent of maximal dose, %	100 (50–100)	100 (50–100)	100 (50–100)	0.933
Diuretics	207 (67.4)	67 (52.8)	140 (77.8)	** < 0.001**
ICD	87 (28.3)	40 (31.5)	47 (26.1)	0.303
CRT-D/CRT-P	83 (27.0)	21 (16.5)	62 (34.4)	**0.001**

*Values are median (interquartile range) or n (%)*

*ACEi/ARB* angiotensin converting enzyme inhibitor / angiotensin II receptor blocker, *BSA* body surface area, *CRT-D* cardiac resynchronization therapy defibrillator, *CRT-P* cardiac resynchronization therapy pacemaker, *ICD* implantable cardioverter defibrillator, *MRA* mineralocorticoid receptor antagonist, *NYHA* New York Heart Association, *PALS* peak atrial longitudinal strain, *percent of maximal dose *= percentage of the maximal recommended dose described in the landmark trials demonstrating survival benefit

Bold *P-*values are statistically significant

**Table 2:** Echocardiographic characteristics at baseline

	**Total population (*n* = 307)**	**PALS > 15% (*n* = 127)**	**PALS ≤ 15% (*n* = 180)**	***P-*value**
**Echocardiographic data**				
LVEDV, mL	169 (134–219)	155 (127–209)	180 (143–223)	**0.002**
LVEDV index, mL/m^2^	93 (75–117)	85 (71–106)	98 (80–123)	**0.007**
LVESV, mL	110 (82–152)	95 (75–134)	125 (93–168)	** < 0.001**
LVESV index, mL/m^2^	62 (45–81)	52 (41–70)	69 (50–88)	** < 0.001**
LVEF, %	35 (27–40)	39 (32–43)	31 (24–38)	** < 0.001**
LVEF < 30%	104 (33.9)	22 (17.3)	82 (45–6)	** < 0.001**
LAVI, mL/m^2^	56 (43–72)	45 (36–59)	63 (50–77)	** < 0.001**
PALS, %	14 (8–20)	22 (18–26)	9 (7–12)	** < 0.001**
EROA, mm^2^	15 (9–22)	13 (7–18)	16 (10–25)	**0.001**
RegVol, mL	25 (14–34)	23 (12–33)	25 (15–35)	0.395
Severe MR	32 (10.4)	7 (5.5)	25 (13.9)	**0.018**
E, m/s	0.87 (0.68–1.05)	0.72 (0.57–0.94)	0.94 (0.76–1.11)	** < 0.001**
E/E’ ratio	13 (10–18)	12 (9–15)	15 (11–19)	** < 0.001**
TR ≥ moderate	55 (17.9)	7 (5.5)	48 (26.7)	** < 0.001**
SPAP, mmHg	41 (33–51)	33 (29–42)	47 (36–56)	** < 0.001**
TAPSE, mm	19 (15–22)	20 (18–22)	17 (14–20)	** < 0.001**

Values are median (interquartile range) or n (%)

*E* peak early diastolic transmitral flow velocity, *E*’ peak early diastolic mitral annular tissue velocity, *EROA* effective regurgitant orifice area, *LAVI* left atrial volume index, *LVEDV* left ventricular end-diastolic volume, *LVEF* left ventricular ejection fraction, *LVESV* left ventricular end-systolic volume, *RegVol* regurgitant volume, *SPAP* systolic pulmonary artery pressure, *TAPSE* tricuspid annular plane systolic excursion, *TR* tricuspid regurgitation. Other abbreviations as in Tables 1

Bold *P*-values are statistically significant

### Prognostic significance of PALS in functional MR

During a median follow-up of 3.5 years (IQR 1.4–5.6), 148 patients (48.2%) died. Of note, during the study period 9 patients performed transcatheter edge-to-edge repair (TEER), 2 underwent surgical mitral valve repair or replacement and 5 patients received heart transplant/LVAD; these patients were right censured at the time of intervention.

**Fig. 2:**
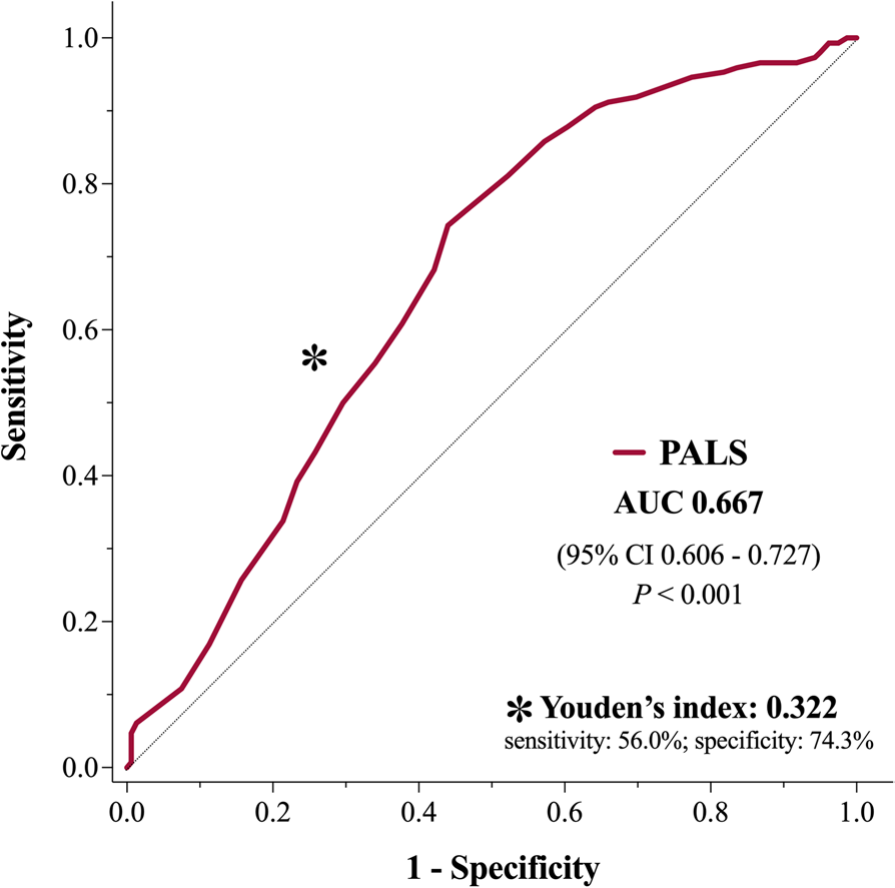
ROC curve for PALS and all-cause mortality. Legend – PALS showed a reasonable discriminative ability for all-cause mortality (AUC 0.667, 95% IC 0.606–0.727, *p* < 0.001). The cut-off of PALS with the highest sensitivity and specificity for all-cause mortality was 15% (Youden’s index 0.322)

PALS showed a reasonable discriminative power for all-cause mortality (C-statistic 0.667, 95% IC 0.606–0.727, *p* < 0.001). The best cut-off of PALS for predicting all-cause mortality was 15% (sensibility 56%, specificity 74.3%, Youden index 0.322), and it was employed to dichotomize the population (Fig. 2). This value was close to the median value of the study population (14%). Overall, 127 (41.4%) patients had PALS > 15% and 180 (58.6%) had PALS ≤ 15%. Detailed baseline clinical and echocardiographic characteristics according to PALS groups are depicted in Tables 1 and 2. Briefly, patients with PALS ≤ 15% were older, more frequently male, had higher prevalence of atrial fibrillation and diabetes, worse NYHA functional class, lower renal function and higher EuroSCORE II. Also, with lower PALS values there were larger indexed left atrial volumes (*P* < 0.001), larger end-systolic LV volumes (*P* < 0.001), higher estimated LV filling pressures (E/E’ ratio, *P* < 0.001), poorer LV and RV function (both *P* < 0.001), and higher EROA and SPAP values (*P* = 0.001 and *P* < 0.001, respectively).

**Fig. 3:**
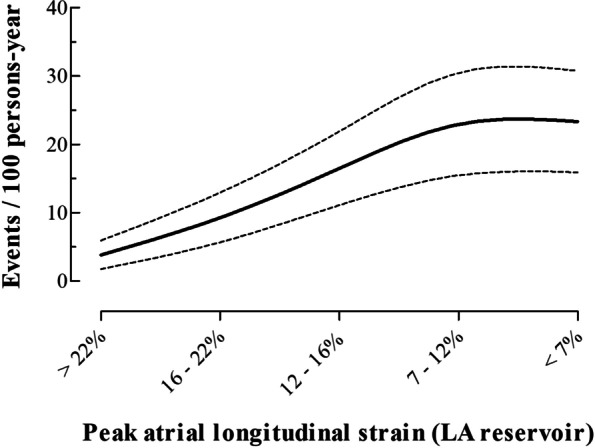
Spline curve for all-cause mortality per 100 persons-year according to PALS. Legend – All-cause mortality per 100 persons-year for progressively lower values of PALS, plotted as a fitted spline model with overlaid confidence intervals. Dashed lines are 95% confidence intervals. Abbreviations as in Fig. 1

**Fig. 4:**
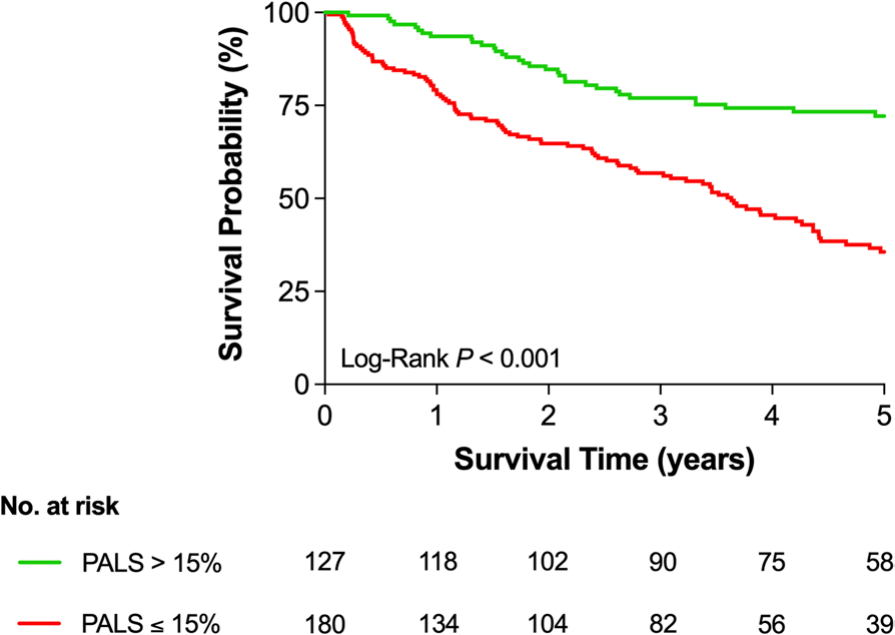
Kaplan–Meier survival curves according to PALS. Legend – Kaplan–Meier survival curves (all-cause mortality) plotted for PALS > 15% (green) and PALS ≤ 15% (red). Overall log-rank *P-*value < 0.001. Abbreviations as in Fig. 1

**Fig. 5:**
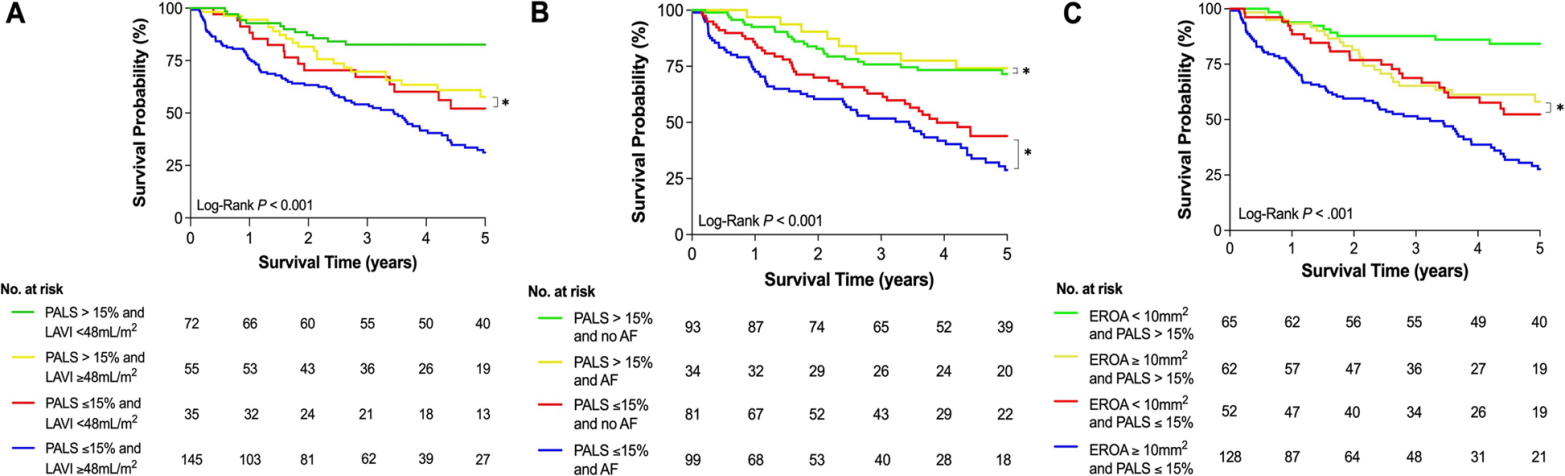
Kaplan–Meier survival curves according to (**A**) PALS and LAVI; (**B**) PALS and history of AF; and (**C**) PALS and EROA. Legend – (**A**) Kaplan–Meier survival curves (all-cause mortality) plotted for each PALS and LAVI group: PALS > 15% and LAVI < 48 mL/m^2^ (green), PALS > 15% and LAVI ≥ 48 mL/m^2^ (yellow), PALS ≤ 15% and LAVI < 48 mL/m^2^ (red), PALS ≤ 15% and LAVI ≥ 48 mL/m^2^ (blue). (**B**) Kaplan–Meier survival curves (all-cause mortality) plotted for each PALS and LAVI group: PALS > 15% and no AF (green), PALS > 15% and AF (yellow), PALS ≤ 15% and no AF (red), PALS ≤ 15% and AF (blue). (**C**) Kaplan–Meier survival curves (all-cause mortality) plotted for each PALS and EROA group: PALS > 15% and EROA < 10mm^2^ (green), PALS > 15% and EROA ≥ 10mm^2^ (yellow), PALS ≤ 15% and EROA < 10mm^2^ AF (red), PALS ≤ 15% and EROA ≥ 10 mm.^2^ (blue). Overall and pairwise log-rank *P-*values were all < 0.05 except for the comparisons highlighted with *. Abbreviations as in Fig. 1

The unadjusted mortality incidence increased with progressively lower values of PALS (Fig. 3), starting at 3.9 events per 100 person-years (95% confidence interval [CI]: 2.2–6.7) in patients with higher PALS values and reaching 23.3 events per 100 person-years (95% CI: 17.5–30.4) in patients with the lowest values of PALS. Kaplan–Meier survival curves were plotted according to the PALS cut-off value of 15% (Fig. 4), confirming a significant increase in mortality with lower PALS values (log rank *P* < 0.001). Additionally, Kaplan–Meier survival curves were also performed when dividing the population into four groups according to both PALS and LAVI, PALS and history of AF, and PALS and EROA (Fig. 5 and Supplementary Table 1).

Cox proportional hazards model was used to investigate the association between PALS and all-cause mortality (Table 3 and Supplementary Table 2). On multivariable analysis, PALS remained independently associated with all-cause mortality (adjusted hazard ratio [aHR]: 1.052 per % decrease; 95% CI: 1.010–1.095; *P* = 0.016). Age (aHR: 1.036 per year; 95% CI: 1.014–1.058; *P* = 0.001), creatinine (aHR: 1.142 per mg/dL; 95% CI: 1.035–1.261; *P* = 0.008), LVEF (aHR: 1.029 per 1% decrease; 95% CI: 1.001–1.059; *P* = 0.046), and EROA (aHR: 1.031 per mm^2^; 95% CI: 1.007–1.056; *P* = 0.012) were the other independent predictors of all-cause mortality. PALS remained independently associated with all-cause mortality after excluding patients with milder degrees of FMR (Supplementary Table 3).

**Table 3:** Univariable and multivariable cox regression analyses

	**Univariable Analysis**			**Multivariable Analysis**		
	**HR**	**95% CI**	***P-*value**	**Adjusted HR**	**95% CI**	***P-*value**
Age, years	1.041	1.024–1.058	** < 0.001**	1.036	1.014–1.058	**0.001**
Male	1.360	0.911–2.032	0.133			
Creatinine, mg/dL	1.106	1.041–1.175	**0.001**	1.142	1.035–1.261	**0.008**
Hypertension	1.685	1.122–2.532	**0.012**	1.261	0.741–2.146	0.394
Diabetes mellitus	1.062	0.748–1.509	0.735			
Atrial fibrillation	1.546	1.119–2.134	**0.008**	1.109	0.714–1.723	0.644
Ischemic etiology	1.104	0.792–1.538	0.558			
NYHA III-IV vs. I-II	1.994	1.430–2.779	** < 0.001**	1.094	0.714–1.676	0.680
Beta-blockers	0.623	0.365–1.063	0.083			
ACEi/ARB	0.577	0.348–0.957	**0.033**	1.820	0.892–3.712	0.100
MRA	0.981	0.709–1.358	0.910			
Diuretics	1.429	1.001–2.040	**0.050**	0.769	0.460–1.186	0.317
LVEDV, mL	1.004	1.001–1.006	**0.002**	1.003	0.999–1.006	0.115
LVEF, per % decrease	0.951	0.934–0.968	** < 0.001**	1.029	1.001–1.059	**0.046**
LAVI, mL/m^2^	1.010	1.006–1.014	** < 0.001**	0.999	0.992–1.007	0.827
PALS, per % decrease	1.080	1.055–1.107	** < 0.001**	1.052	1.010–1.095	**0.016**
EROA, mm^2^	1.042	1.028–1.057	** < 0.001**	1.031	1.007–1.056	**0.012**
SPAP, mmHg	1.031	1.020–1.042	** < 0.001**	1.011	0.995–1.027	0.193
TAPSE, mm	0.931	0.896–0.967	** < 0.001**	1.021	0.973–1.071	0.405
TR ≥ moderate	2.180	1.491–3.188	** < 0.001**	0.887	0.556–1.415	0.614

*CI* confidence interval, *HR* hazard ratio; other abbreviations as in Tables 1 and 2

## Discussion

We report the prognostic significance of PALS in a population with reduced LVEF and across the whole spectrum of ventricular FMR, as assessed by all-cause mortality. Our main findings were as follows: (1) Impaired PALS is associated with poorer LV function and more severe FMR; and (2) In patients with reduced LVEF and FMR, PALS is independently associated with all-cause mortality and holds additional information over conventional LA and LV measurements.

### LA reservoir function in functional MR

Ventricular FMR is a frequent finding in HF patients and is associated with worse prognosis, in part related to the additional volume overload and increased LV end-diastolic wall stress and remodeling [26]. However, it was not up until recently it was recognized the key role of LA in the pathophysiology: it is a major determinant of LV filling, operates as a watershed between the LV and the pulmonary circulation, and holds important endocrine functions [6–8, 27]. Mitral regurgitation-related chronic volume overload induces significant LA structural and histological changes (including dilatation and interstitial fibrosis), leading to a marked reduction in LA compliance over time [28]. In this regard, LAVI has been consistently reported as a marker of adverse outcomes in a variety of cardiovascular diseases, including mitral regurgitation [29]. Nonetheless, previous reports have suggested that LA phasic function parameters (particularly PALS) can identify atrial dysfunction at earlier stages, even before dilatation occurs [9, 30]. On the other hand, while LA size is perceived as a marker of a chronically elevated LA pressure, its function (assessed by means of LA strain) represents dynamic loading conditions and can therefore represent a more accurate assessment of LV filling pressures [31]. We showed that patients with impaired PALS were more likely to present with higher degrees of LV remodeling (lower LVEF and higher LV volumes) and of FMR severity. These findings may reflect the extent of ultrastructural and functional atrial changes noticed in more advanced stages of the disease, as previous works demonstrated a close relationship of PALS with LA fibrosis and dysfunction [19].

A study reporting a very similar PALS cut-off to the one we have found (14% vs. 15%) has demonstrated the interplay between FMR and LA function [32]. Not only patients with higher degrees of FMR presented with lower values of PALS, as PALS was found to modulate the prognostic impact of FMR. Indeed, in any FMR grade, clinical features of severity and dismal outcomes were observed in patients with reduced LA function (defined as PALS < 14%), whereas those with preserved reservoir function presented with a more benign clinical course [32]. In both scenarios, FMR with EROA ≥ 0.3 cm^2^ was associated with worse prognosis independently of PALS. Beyond modulating ventricular FMR prognosis, progressively lower PALS were also found to have a linear relationship with worse outcomes in patients with HF with reduced LVEF, with excess risk occurring for a PALS value inferior to the previously identified threshold [33].

### Prognostic significance of PALS in FMR and clinical implications

Globally, patients with HF and FMR have an extremely poor prognosis with reported mortality rates of up to 47% at 5 years of follow-up, although it can vary significantly according to individual clinical variables [26]. Identifying these features might be of interest for better risk stratification and evidence-based clinical decision-making. Despite the prognostic significance of PALS having been previously reported in patients with primary mitral regurgitation, it was not extensively studied in the setting of FMR in patients with LV systolic dysfunction. In our cohort, we found higher mortality incidence for progressively lower values of PALS and that a threshold of ≤ 15% was the best to predict mortality. This cut-off was higher than the one proposed by Stassen et al., that is 9.8% [27]. In fact, in our cohort only 10% had severe FMR, but, contrarily to Stassen et al. [27], we included patients with at least mild FMR. Accordingly, we tested the prognostic significance of PALS across the continuum of ventricular FMR severity, as previous studies have shown that even mild degrees of FMR associate with worse outcomes in patients with chronic HF and reduced LVEF [26, 32]. PALS retained incremental prognostic significance over FMR severity, LAVI and other clinical and echocardiographic variables. As such, the addition of PALS, but not of LAVI, to a clinical multivariable model improved its ability to predict all-cause mortality. Yet, care is advised when interpreting PALS, as 2D speckle tracking echocardiography-derived strain values can vary significantly among different vendors [12].

These present data show that PALS can be a promising index, as it holds independent prognostic significance across the whole spectrum of ventricular FMR, including mild regurgitation. In fact, the paradigm in the management of valvular heart disease is shifting towards the use of more advanced imaging measurements such as speckle tracking echocardiography (including LV global longitudinal strain [GLS]) and tissue characterization with late gadolinium enhancement cardiac magnetic resonance (CMR) [34]. These early markers of myocardial damage have shown incremental prognostic significance when compared to LVEF alone and may be important in identifying patients that would benefit from prompt referral for intervention [34]. In the particular setting of FMR, and although further studies are necessary to validate these results, PALS might be of value in the follow-up. Representing an emerging imaging marker of early cardiac damage, in selected cases (e.g., moderate to severe FMR), it may be useful in identifying higher-risk subgroups for patient-tailored treatment guidance.

### Limitations

Some limitations of this study should be acknowledged. Because of the retrospective nature of the study, a significant proportion of patients initially screened was excluded due to suboptimal frame rate or inadequate acoustic window quality, which is known to increase the risk of bias (Supplementary Fig. 1). Secondly, in our cohort, we also included patients with mild FMR and only about 10% had severe FMR, which may pose some issue in generalizing the results for patients with significant (i.e., at least moderate) FMR. Thirdly, mortality was ascertained through a national database, and therefore it was not possible to distinguish cardiovascular and non-cardiovascular causes of death. Although we have proposed a PALS cut-off of ≤ 15% to predict higher mortality in this population, the absence of a validation cohort should be stated as a limitation. External validation is necessary to confirm these results. Finally, we did not perform LV GLS analysis due to inadequate image quality in many patients. To tackle this limitation, we calculated LV GLS in 42 random patients with reduced LVEF and FMR and found a strong linear inverse correlation with PALS (Pearson Correlation -0.706, *p* < 0.001) (Supplementary Fig. 2). In fact, LV GLS is known to be one of the most important, but not the sole, determinants of LA reservoir function [35, 36]. Although some authors even suggest that it contains little added information to LV GLS, we provide evidence that PALS is a reproducible measurement and holds a robust prognostic significance in patients with LV dysfunction and FMR [35]. Moreover, PALS can be easily acquired in an apical 4-chamber view alone and may be easier to obtain in patients with poor acoustic windows and/or in the presence of atrial fibrillation, in whom LV GLS measurement from all three apical projection may not be feasible.

## Conclusion

In a cohort of patients with reduced LVEF and FMR, impaired PALS was independently associated with an increased risk for all-cause mortality. PALS assessment may be an easy and emerging method for prognostic purposes and therapeutic guidance in this population.

## Supplementary Information

**Additional file 1.**

## Data Availability

Data will be shared on reasonable request to the corresponding author.
