# Supplementary material for: Peak left atrial longitudinal strain is associated with all-cause mortality in patients with ventricular functional mitral regurgitation
Source: Cardiovasc Ultrasound. 2023 May 6;21:9. doi: 10.1186/s12947-023-00307-7 (PMC10163691; doi:10.1186/s12947-023-00307-7)

[SUPPLEMENTARY MATERIAL]

Supplementary Figure 1

Title – Study Flowchart

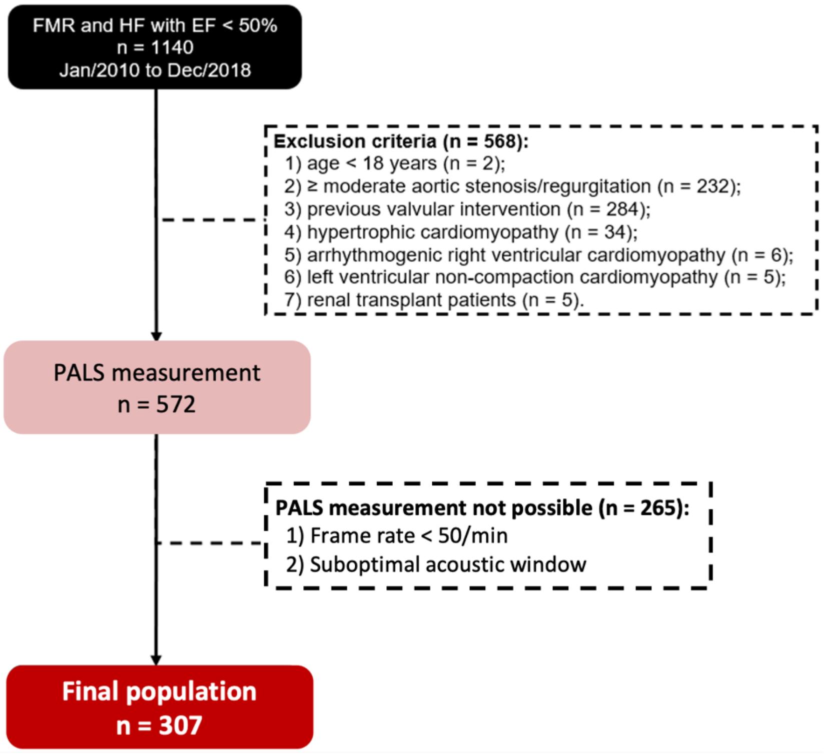

Supplementary Table 1

Title – Pairwise Log-Rank P-values for PALS and LAVI, PALS and history of AF, and PALS and EROA categories.

Legend – Pairwise comparison for PALS and LAVI, PALS and history of AF, and PALS and EROA categories. Groups for each category and abbreviations as in **Figure 5**.

	PALS and LAVI (P-value)				***PALS and history of AF (P-value)***				***PALS and EROA (P-value)***			
	Group 1	***Group 2***	***Group 3***	***Group 4***	***Group 1***	***Group 2***	***Group 3***	***Group 4***	***Group 1***	***Group 2***	***Group 3***	***Group 4***
Group 1	-	*0.003*	*<0.001*	*<0.001*	-	*0.04*	*<0.001*	*<0.001*	-	*<0.001*	*<0.001*	*<0.001*
Group 2	-	-	*0.343*	*<0.001*	-	-	*0.002*	*<0.001*	-	-	*0.642*	*<0.001*
Group 3	-	-	-	*0.041*	-	-	-	*0.173*	-	-	-	*<0.001*

PALS and LAVI	PALS and history of AF	PALS and EROA
Group 1 – PALS > 15% and LAVI < 48mL/m2	*Group 1 – PALS > 15% and no AF*	*Group 1 – PALS > 15% and EROA < 10mm2*
Group 2 – PALS > 15% and LAVI ≥ 48mL/m2	*Group 2 – PALS > 15% and AF*	*Group 2 – PALS > 15% and EROA ≥ 10mm2*
Group 3 – PALS ≤ 15% and LAVI < 48mL/m2	*Group 3 – PALS ≤ 15% and no AF*	*Group 3 – PALS ≤ 15 % and EROA < 10mm2*
Group 4 – PALS ≤ 15% and LAVI ≥ 48mL/m2	*Group 4 – PALS ≤ 15% and AF*	*Group 4 – PALS ≤ 15% and EROA ≥ 10mm2*

Supplementary Table 2

Title – Cox proportional hazard model for all-cause mortality

Legend – PALS remained independently associated with all-cause mortality in a Cox proportional hazard model with progressively more clinical and echocardiographic variables. Abbreviations as in **Tables 1** and **2**.

	**Adjusted HR**	**95% CI**	**P-value**
Unadjusted	1.080	1.055–1.107	<0.001
Adjusted for age, sex, and creatinine	1.070	1.042–1.099	<0.001
Adjusted for sex, age, creatinine, LVEF and EROA	1.044	1.012–1.107	0.006
Adjusted for multiple clinical and echocardiographic variables *	**1.052**	**1.010–1.095**	**0.016**

** Ajusted for sex, age, creatinine, LVEF, EROA, arterial hypertension, atrial fibrillation, NYHA, ACEi/ARB, diuretics, LVEDV, LAVI, SPAP, TAPSE, at least moderate tricuspid regurgitation*

Supplementary Table 3

Title – Multivariate Cox regression sensitivity analysis excluding patients with milder degrees of FMR (EROA < 0.1cm^2)

Legend – After excluding patients with milder FMR (EROA < 0.1cm^2), decreased values of PALS remained significantly associated with all-cause mortality. Abbreviations as in **Tables 1** and **2**.

	**Multivariable Analysis**		
	**Adjusted HR**	**95% CI**	**P-value**
Age, years	1.032	1.006–1.059	**0.015**
Creatinine, mg/dL	1.101	0.978–1.239	0.113
Hypertension	1.067	0.514–2.217	0.861
Atrial fibrillation	1.376	0.825–2.296	0.222
NYHA III/IV vs. I/II	0.968	0.590-1.588	0.896
ACEi/ARB	2.237	0.986–5.076	0.054
Diuretics	0.597	0.324–1.098	0.097
LVEDV, mL	1.004	1.000–1.009	**0.040**
LVEF, %	0.971	0.939–1.004	0.080
LAVI, mL/m^2	0.997	0.988–1.006	0.484
PALS, per % decrease	1.050	1.001-1.103	**0.047**
EROA, mm^2	1.029	0.999–1.060	0.062
SPAP, mmHg	1.010	0.992–1.028	0.268
TAPSE, mm	1.028	0.971–1.089	0.346
TR ≥ moderate	0.820	0.499–1.348	0.434

Supplementary Figure 2

Title – Correlation between PALS and GLS in patients with FMR

Legend – Pearson correlation analysis showing a strong linear inverse correlation between PALS and GLS (r = -0.706, p<0.001). Abbreviations as in **Figure 1**.

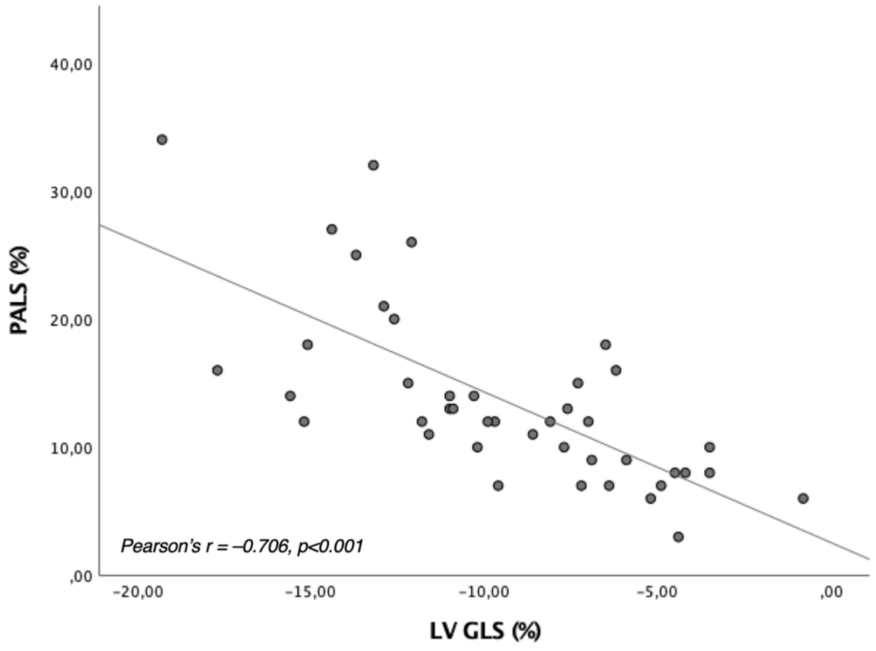

Supplement: Supplementary file 1 — Additional file 1. [file 12947_2023_307_MOESM1_ESM.pdf]
